# Multisensory perception and action: development, decision-making, and neural mechanisms

**DOI:** 10.3389/fnint.2013.00081

**Published:** 2013-11-21

**Authors:** Zhuanghua Shi, Hermann J. Müller

**Affiliations:** ^1^Department of Psychology, Experimental Psychology, Ludwig-Maximilians-Universität MünchenMunich, Germany; ^2^Department of Psychological Science, Birkbeck College, University of LondonLondon, UK

**Keywords:** multisensory perception, multisensory timing, multisensory development, multisensory learning, multisensory neural mechanisms

Surrounded by multiple objects and events, receiving multisensory stimulation, our brain must sort through relevant and irrelevant multimodal signals to correctly decode and represent the information from the same and different objects and, respectively, events in the physical world. Over the last two decades, scientific interest has increased dramatically in how we integrate multisensory information and how we interact with a multisensory world, as evidenced by exponential growth of the relevant studies using behavioral and/or neuro-scientific approaches.

The Special Issue topic of “Multisensory perception and action: psychophysics, neural mechanisms, and applications” emerged from a scientific meeting dedicated to these issues: the *Munich Multisensory Perception Symposium* held in Holzhausen am Ammersee, Germany (June 24–26, 2011). This volume, which collects research articles contributed by attendees of the symposium as well as the wider community, is organized into three interrelated sections:

- Development, learning, and decision making in multisensory perception
- Multisensory timing and sensorimotor temporal integration
- Electrophysiological and neuro-imaging analyses of multisensory perception

## Development, learning, and decision-making in multisensory perception

Many multisensory studies, ranging from spatial (e.g., Ernst and Banks, 2002; Alais and Burr, 2004) to temporal integration (e.g., Burr et al., 2009; Chen et al., 2010; Shi et al., 2013b), reveal that our brain combines multisensory signals if they are closely relevant to the task, in order to boost overall performance. Senses, however, are not the only source for decision-making. Prior, contextual, and symbolic cues can also contribute as an extra source of information to improve performance (Jazayeri and Shadlen, 2010; Petzschner and Glasauer, 2011; for a review, see Shi et al., 2013a). Accordingly, Petzschner et al. (2012) set out to examine how auxiliary contextual cues, such as symbolic “short” and “long” cues, are used optimally in a distance production-reproduction task. Their findings indicate that humans are capable of using symbolic cues for final estimates, even though the mapping of the symbolic cue onto the stimulus dimension has to be learned during the experiment.

With respect to learning, one prominent question in multisensory integration concerns when and how we acquire the capacity to optimally integrate multisensory cues. Some recent studies suggest that this capacity is not present at birth, but rather develops after about 8 years of age (e.g., Gori et al., 2008). Gori et al. (2012) expanded this line of research by examining audio-visual temporal and spatial bisection tasks in young children, finding that young children exhibit strong unisensory dominance over multisensory integration of audiovisual signals, with audition dominating audiovisual time perception and vision dominating space perception. Both dominance effects reflect a process of cross-sensory calibration of developing systems, where the more accurate sense calibrates or teaches the other, rather than fusing with it. In another study, Wismeijer et al. (2012) showed that our brain also exhibits remarkable ability to learn cue-associations, such as an arbitrary association of visual gloss and touch softness, and use the learned associate-cues for judgments—with learning being more efficient from touch-to-vision than from vision-to-touch, which is in line with earlier evidence of touch teaching vision for size discrimination in young children (Gori et al., 2008).

Multisensory signals, compared to separate unisensory signals, not only enhance overall performance, but also facilitate the speed of responses. Based on their previously developed framework of the time-window-of-integration (TWIN), Colonius and Diederich (2012) provided further qualitative and quantitative predictions of the TWIN model regarding how the probability of multisensory integration would affect response facilitation differently in the crossmodal-signals and the focused-attention paradigm. In the reverse direction Hong et al. (2012) examined response impairments arising from conflicting crossmodal stimuli or configurations that engender multisensory illusions, in particular, the hand-reversal illusion.

## Multisensory timing and sensorimotor temporal integration

Time perception is susceptible to a wide range of factors (Shi et al., 2013a), in particular with multisensory inputs. A number of authors examining this set of issues have attempted to pin down key factors in multisensory timing. With regard to the perception of multisensory durations, Shi et al. (2012) showed that high-arousal affective pictures have differential impacts on subsequent tactile duration judgments, with pictures that evoke threat meanings expanding subjective duration, whereas pictures that evoke disgust meanings exhibiting no effects on tactile temporal judgments—indicative of the importance of crossmodal connections in the processing of multisensory timing. Ganzenmüller et al. (2012) further demonstrated that delayed onset of auditory signals generated by participants' manual button press immediately lengthens the reproduced duration, whereas offset delays did not—showing that multisensory timing relies differentially on sensory and motor signals in duration reproduction. Using apparent motion as an implicit measure of perceived duration, Zhang et al. (2012) reported another differential adaptation effect in multisensory timing: adaptation to a short auditory or visual interval resulting in a consistent negative aftereffect for Ternus apparent motion, whereas adaptation to a long interval yielded an aftereffect only for the auditory, and not the visual, condition.

Similar to multisensory duration, multisensory temporal-order processing is also influenced by many factors. For example, to identify key physical changes associated with the articulation of consonants and vowels that may influence the temporal integration window for audiovisual speech, Vatakis et al. (2012) examined the perception of audiovisual synchrony using video clips uttered by different speakers with differential audiovisual signal saliencies (with auditory saliency measured by a combination of three acoustic features: instantaneous energy of the most active filter, instantaneous amplitude, and frequency of the dominant filter's output; and visual saliency computed by intensity, color, and motion). They found that the (degree of) saliency of visual-speech signals can modulate the lead of visual over auditory signals that is necessary for them to be perceived as simultaneous, the lead typically found in audiovisual speech perception. These findings thus support the “information reliability hypothesis,” on which the perception of a multisensory feature is dominated by the modality that provides the most reliable information (Welch and Warren, 1980; Ernst et al., 2004). Similarly, Hendrich et al. (2012) found that not only stimuli features, but also task requirements, such as dual tasks, could affect audio-visual temporal-order judgments, arguing that the influence of dual tasks on crossmodal temporal processing is mainly on the perceptual, rather than the response-selection, stage.

## Electrophysiological and neuro-imaging analyses of multisensory perception

The neural mechanisms underlying integrative and interactive functions are central to understanding multisensory perception. Quite a number of studies concerned with these functions have been designed to elucidate how information that comes from different sensory modalities are processed and integrated in the brain.

Several studies provide found evidence that multisensory signals are integrated at a very early stage. Naci et al. (2012), for example, found that higher-order regions in anterior temporal (AT) and inferior prefrontal cortex (IPC) performed audio-visual integration 100 ms earlier than a sensory-driven region in the posterior occipital (pO) cortex, suggesting the brain represents familiar and complex multisensory objects through early interactivity between higher-order, and sensory-driven regions. Stekelenburg and Vroomen (2012) also showed that spatial congruity between auditory and visual signals modulates audiovisual interactions reflected in early ERP components, namely, the N1 and P2. Early integration may boost the saliency of the multisensory signals, even when the multisensory signals are irrelevant distractors, causing an attentional shift toward the multisensory distractor, as measured by steady-state visual evoked potentials (SSVEP) in an audiovisual speech task (Krause et al., 2012). Instead of using multisensory signals, Töllner et al. (2012) presented separate auditory and visual signals in a dual-task paradigm requiring both auditory and visual discriminations, to investigate influences of task order predictability (TOP) and inter-task onset asynchrony (SOA) on perceptual, and motor processing stages, two stages indexed, respectively, by two EEG components: the Posterior-Contralateral- Negativity (PCN) and the Lateralized-Readiness-Potential (LRP). Töllner et al. found TOP to interact with inter-task SOA in determining the speed of perceptual processing, providing electrophysiological evidence of central capacity limitations in the processing of auditory and visual dual tasks.

Using functional MRI imaging techniques, two other studies examined brain regions involved in multisensory perception. Noesselt et al. (2012) investigated the neural basis of the perception of synchrony/asynchrony for audiovisual speech stimuli, and found a distinct pattern of modulations within the multisensory superior temporal sulcus complex (mSTS-c): “auditory leading (AL)” and “visual leading (VL) areas” lie closer to “synchrony areas” than to each other, suggesting the presence of distinct sub-regions within the human STS-c for the maintenance of temporal relations for audiovisual speech stimuli, with differential functional connectivity with prefrontal regions. Beer et al. (2013), on the other hand, found bimodal presentation of audiovisual speech and audiovisual movement stimuli, compared to unimodal stimulation, engaged a temporal-occipital brain network including the multisensory superior temporal sulcus (msSTS), the lateral superior temporal gyrus (ISTG), and the extrastriate body area (EBA). Moreover, brain areas involved in multisensory processing showed little direct connectivity with primary sensory cortices; rather these brain areas were connected to early sensory cortices via intermediate nodes of the STS and the inferior occipital cortex (IOC).

Taken together, this collection provides a broad-spectrum but overall coherent addition to the rapidly growing field of multisensory perception and action. Of course, more work needs to be carried out and many open questions and issues (some of which are identified in the present collection) remain to be addressed in order to achieve a full understanding the functions and neural mechanisms of multisensory perception and action. We would like to thank all the authors, the expert reviewers, and the Frontiers staff for helping to make this Special Issue possible. We hope this collection can act as a catalyst for some of the future work, and we look forward to further explorations of multisensory perception and action.
